# Evaluating the Efficacy of a Semi-Solid Formulation with Clove Oil and Curcumin versus Clindamycin in the Treatment of Acne Vulgaris: A Comprehensive Study of Preclinical and Clinical Findings

**DOI:** 10.34172/apb.025.45153

**Published:** 2025-08-30

**Authors:** Peymaneh Dastgir, Setareh Tehrani, Setareh Haghighat, Sepideh Arbabi Bidgoli, Solmaz Ghaffari

**Affiliations:** ^1^Pharmaceutical Sciences Research Center, TeMS.C., Islamic Azad University, Tehran, Iran; ^2^Chair of the Department of Dermatology, TeMS.C., Islamic Azad University, Tehran, Iran; ^3^Department of Microbiology, TeMS.C., Islamic Azad University, Tehran, Iran; ^4^Department of Toxicology and Pharmacology, TeMS.C., Islamic Azad University, Tehran, Iran; ^5^Department of Pharmaceutics, TeMS.C., Islamic Azad University, Tehran, Iran

**Keywords:** Acne vulgaris, Natural ingredients, Clindamycin, Semi-solid formulation, Clinical study, Side effects

## Abstract

**Purpose:**

Acne vulgaris is a chronic inflammatory condition affecting the pilosebaceous units. With the rise of antibiotic resistance and the potential side effects associated with conventional treatments, there is increasing interest in exploring natural alternatives for treating acne. This study aimed to formulate and clinically evaluate a topical gel containing clove oil and curcumin in patients with mild to moderate acne vulgaris, using topical clindamycin as a standard comparator.

**Methods:**

The antibacterial activity of clove oil and curcumin against *Propionibacterium acnes* and *Staphylococcus epidermidis* was assessed by determining their minimum inhibitory concentration (MIC) and minimum bactericidal concentration (MBC). Based on these findings, semisolid gel formulations were developed and subjected to in vitro evaluations. Subsequently, a single-blind, randomized, comparative clinical study was conducted in 31 participants diagnosed with mild to moderate acne. Volunteers applied clindamycin gel in the morning and the clove oil–curcumin gel in the evening, or vice versa, over a 4-week period. Clinical outcomes, including acne lesion counts, Acne Severity Index (ASI), and patient satisfaction, were assessed.

**Results:**

The clove oil-curcumin gel demonstrated comparable efficacy to clindamycin in reducing acne lesions, papules, and ASI. While no significant differences were observed between the two groups in comedone reduction, patient satisfaction increased in clove oil-curcumin gel group.

**Conclusion:**

Topical application of a gel containing clove oil and curcumin demonstrated promising results as an effective and well-tolerated alternative or adjunctive therapy for acne vulgaris. These findings support the potential of plant-based formulations in acne management.

## Introduction

 Acne vulgaris is a prevalent chronic skin condition associated with inflammation of the pilosebaceous units. It typically presents as non-inflammatory and inflammatory lesions, such as comedones, papules, pustules, nodules, and cysts, and may result in permanent scarring.^1^ The disease develops through a multifactorial process that includes excessive sebum secretion, abnormal keratinization within hair follicles, bacterial colonization particularly by *Propionibacterium acnes* and *Staphylococcus epidermidis* and subsequent inflammatory responses.^1,2^

 The psychological burden of acne is substantial, as it can lead to emotional distress, reduced self-confidence, social isolation, and in severe cases, anxiety, depression, or suicidal thoughts.^3-5^ Therefore, effective treatment strategies are not only aimed at lesion reduction but also at improving patients’ quality of life.

 Currently available treatments comprise topical agents (e.g., benzoyl peroxide, tretinoin, azelaic acid and topical antibiotics e.g., clindamycin) and systemic medications (e.g., oral antibiotics, isotretinoin).^6^ Despite their clinical efficacy, these therapies often present drawbacks, including microbial resistance, gastrointestinal side effects, photosensitivity, and potential teratogenicity, especially with long-term use.^7-10^ As a result, there is increasing interest in the development of safer, naturally derived alternatives with fewer side effects.

 Although various herbal agents have been traditionally used for acne, standardized and officially approved natural products remain scarce in many countries. This prompted research into natural formulations including herbals that may offer therapeutic benefits with enhanced safety profiles.

 The current study explores the formulation of two semisolid topical preparations containing natural active pharmaceutical ingredients (APIs) with established antimicrobial and anti-inflammatory properties: clove oil and curcumin.

 Clove oil, obtained from *Syzygium aromaticum* showed broad-spectrum antimicrobial activity, particularly against acne-associated bacteria. It also exhibits anti-inflammatory and antioxidant activities, so its potential of dermatological applications was stablished previously.^11-16^

 Curcumin, a polyphenol derived from the rhizome of *Curcuma longa*, exhibits antimicrobial, anti-inflammatory, and antioxidant properties. It inhibits the growth of *P. acnes* and *S. epidermidis*, suppresses the production of inflammatory cytokines by *P. acnes*, and promotes wound healing and scar reduction.^17-28^

 This study aimed to assess the feasibility of incorporating these two natural agents into topical formulations for the management of mild to moderate acne, potentially contributing to the development of safer and more acceptable treatment options as well as reducing the psychological effects of acne in patients.

## Material and Methods

###  Materials

 Tween 80, Span 80, propylene glycol, methylparaben, propylparaben, and triethanolamine were purchased from Merck (Germany). Microbial culture media were obtained from Merck (Germany) and QUELAM. Curcumin was sourced from Sami Lab (India), and clove oil was provided by Ghatran Shimi Co. (Iran). Standard strains of *P. acnes* ATCC 6919 and *S. epidermidis* ATCC 12228 were obtained from the Pasteur Institute of Iran’s microbial culture collection. Cultures were maintained on appropriate media under standard conditions.

###  Antimicrobial susceptibility testing

####  Disk diffusion method

 Bacterial suspensions (1.5 × 10⁸ CFU/mL) of *P. acnes* and *S. epidermidis* were prepared and inoculated on blood agar and Mueller-Hinton agar plates, respectively. Sterile filter paper discs were impregnated with various concentrations of clove oil and curcumin solutions for 1 hour and then placed on the inoculated plates. Pre-diffusion was allowed for 30 minutes at room temperature. Plates were incubated at 37 °C for 96 hours for *P. acnes* and 20 hours for *S. epidermidis*. Standard clindamycin discs (2 μg/disc) served as positive controls. Inhibition zones were measured in millimeters. All experiments were performed in triplicate.^28^ Supplementary file, Figure S1 shows inhibition zones.

####  Agar well diffusion method

 Bacterial suspensions (1.5 × 10⁸ CFU/mL equal to 0.5 McFarland) were prepared. Suspensions were swabbed onto the surface of blood agar plates for *P. acnes* and Mueller-Hinton agar plates for *S. epidermidis*. Clove oil solutions in ethanol were prepared at concentrations ranging from 0.0625 to 4% (v/v) for *S. epidermidis* and 0.03125 to 2% (v/v) for *P. acnes*. Curcumin solutions in ethanol were prepared at concentrations ranging from 1000 to 31.25 μg/mL. Wells were created in the agar plates. Solutions were added to the respective wells. Plates were pre-incubated for 30 minutes. Plates were incubated at 37 °C for 20 hours for *S. epidermidis* and 96 hours for *P. acnes*. Zones of inhibition were measured.^29^

####  Broth micro dilution method

 The minimum inhibitory concentrations (MICs) and minimum bactericidal concentrations (MBCs) of clove oil and curcumin were determined using the broth micro dilution method in sterile 96-well microplates.

####  Experimental procedure

 Each well was filled with 100 µL of Mueller-Hinton broth for *S. epidermidis* and Brain Heart Infusion (BHI) broth for *P. acnes*. Stock solutions of clove oil and curcumin were added to the first wells and subjected to eight-fold serial dilutions across the plate. Bacterial suspensions were adjusted to 0.5 McFarland standard (~1.5 × 10⁸ CFU/mL), and 60 µL of each suspension was added to the wells, resulting in a final bacterial concentration of approximately 1.5 × 10⁸ CFU/mL per well. Positive and negative controls were included in each assay. The negative control consisted of clindamycin and broth media (no bacterial growth), while the positive control consisted of bacterial suspension and media without antimicrobial agents. The plates were incubated at 37 °C under anaerobic conditions for 96 hours for *P. acnes* and under aerobic conditions for 24 hours for S. epidermidis. All tests were performed in triplicate.

####  MIC determination

 The MIC was defined as the lowest concentration of the test compound that completely inhibited visible bacterial growth.

####  MBC determination

 Aliquots (10 µL) from wells at and above the MIC level were sub cultured on appropriate agar plates and incubated under the same conditions described above. The MBC was defined as the lowest concentration of the test agent resulting in no visible bacterial growth on the agar surface. All experiments were conducted in triplicate.

###  Antibacterial combination study by checkerboard method

 The checkerboard microdilution method was employed to determine the FIC indices of the active ingredients.

####  Experimental procedure

 After determining the individual MICs of each active ingredient, their combined effects were evaluated using the microdilution method. 100 μL of selective broth media and 60 μL of bacterial inoculum (1.5 × 10^8^ CFU/mL) were added to each microtiter plate well. Two-fold serial dilutions of the active ingredients were prepared in combination (1:1 v/v), and 100 μL of each combination was added to the wells. Incubation conditions were maintained as previously described for individual MIC determinations.

####  FIC index calculation

 The FIC index was calculated using the following formula:

*FIC index=(MIC of EOA in combination / MIC of EOA alone)+(MIC of EOB in combination / MIC of EOB alone)*

 Where EOA and EOB represent the two different essential oils being tested.

 FIC indices were interpreted as follows:

- FICI ≤ 0.5: Synergistic interaction
- 0.5 < FICI ≤ 4: Additive interaction
- FICI > 4: Antagonistic interaction

All experiments were performed in triplicate.

###  Formulation of gels

 Tween 80, ethanol, and propylene glycol were used as co-solvents for clove oil, while propylene glycol was selected as the levigating agent for curcumin. The composition of the investigated formulations is summarized in Table 1. Carbopol 940 was dispersed in distilled water and stirred at 200–300 rpm for 2 hours. Clove oil and curcumin were levigated with titanium dioxide (used as a masking agent) in propylene glycol. This mixture was subsequently dissolved in ethanol containing 0.25% (w/v) methylparaben and 0.05% (w/v) propylparaben as preservatives. The resulting solution was added to the previously prepared carbomer dispersion and stirred for an additional 10 minutes at 200–300 rpm. The final pH of the formulation was adjusted to 4–6 using triethanolamine.

**Table 1:** Formulation composition of gels and emulgels

Formulation*	**TiO**_2_	**Carbopol % (w/w)**	**Ethanol**	**Propylene glycol % (w/w)**	**Tween 80**	**LLP**	**Span**
**Formulation composition of gels**							
A1	0.5	10	5	2	5	-	-
A2	0.6	10	5	2	5	-	-
A3	0.75	10	5	2	5	-	-
A4	1	10	5	2	5	-	-
B1	0.6	10	7.5	2	2.5	-	-
B2	0.6	20	7.5	2	2.5	-	-
B3	0.6	30	7.5	2	2.5	-	-
C1	0.6	30	7.5	2	5	-	-
C2	0.6	30	10	2	5	-	-
**Formulation composition of emulgels**							
D1	5	0.5	7.5	0.84	10	2.16	5
D2	5	0.6	7.5	0.84	10	2.16	5
D3	5	0.75	7.5	0.84	10	2.16	5
D4	5	1	7.5	0.84	10	2.16	5
E1	7.5	0.6	7.5	0.84	10	2.16	2.5
E2	10	0.6	7.5	0.84	10	2.16	2.5
F1	10	0.6	7.5	0.84	10	2.16	5
F2	10	0.6	10	0.84	10	2.16	5

###  Formulation of emulgel

 The detailed composition of the investigated emulgel formulations is presented in Table 1. The gel base was prepared by dispersing Carbopol 940 in purified water with continuous stirring, followed by neutralization with triethanolamine to adjust the pH to 4–6. The oil-in-water (O/W) emulsion was prepared by separately heating and mixing the oil and aqueous phases. Oil phase was prepared by mixing of light liquid paraffin (LLP) with appropriate amounts of Tween 80 and Span 80. The surfactant ratio was determined based on the required HLB value. For preparing of aqueous phase, methylparaben and propylparaben were dissolved in purified water. Both phases were heated to 75 ± 1 °C and 80 ± 1 °C, respectively. The aqueous phase was added dropwise to the oil phase under continuous stirring, and the emulsion was allowed to cool gradually to room temperature. Clove oil, curcumin, and titanium dioxide were levigated in propylene glycol and dissolved in ethanol. This solution was then incorporated into the emulsion. Finally, the prepared emulsion was blended with the gel base in a 1:1 ratio under continuous stirring to obtain the final emulgel.

###  In-vitro evaluation of prepared formulations

####  Physical appearance

 All prepared gel and emulgel formulations were evaluated for color, homogeneity, consistency, grittiness, and phase separation conditions.^30-32^

####  Spreadability assessment

 The formulations were manually applied to the back of the hand to assess spread ability. Formulations demonstrating uniform distribution, ease of application, and absence of grittiness were considered acceptable.

####  Quantitative measurement

 An accurately weighed amount (0.5 g) of each sample was placed between two glass slides. A 500 g weight was applied for 1 minute to achieve uniform thickness. The diameter of the spread was measured, and spread ability was calculated based on the circular area.^33^

####  Stress stability test

 The mechanical stability of the emulgel was evaluated through centrifugation under different rpms and times. After each centrifugation cycle, the formulations were visually inspected for phase separation. Stable emulgels were defined as those exhibiting no signs of creaming, cracking, or phase separation during or after the test.^34^

####  Determination of emulgel type (O/W or W/O)

 Water wash test: A small amount of emulgel was placed on the hand and washed with water. Ease of removal with water typically indicates an O/W emulsion, where water constitutes the continuous external phase.

####  Methylene blue dye test

 Methylene blue dye is water-soluble and exhibits a blue color in aqueous solutions. For this test, a small amount of methylene blue dye was added to the emulgel. The appearance of a blue color within the emulgel suggests an O/W emulsion.

####  pH determination

 The pH of all formulations was determined using calibrated pH-meter. Each formulation consists of water (propyl paraben 0.02%w/w and methyl paraben 0.18% w/w). All the formulations were neutralized by triethanolamine to pH = 4-6.

####  Viscosity measurement and rheological behavior studies

 The viscosity and rheological behavior of the final formulations were evaluated using a Brookfield viscometer equipped with spindle 52.

 0.5 g of each gel sample was placed on the viscometer plate. The cone spindle was carefully lowered onto the sample. Viscosity measurements were conducted at room temperature. The rotational speed (rpm) was varied, and the corresponding viscosity, shear stress, shear rate, and torque values were recorded. A plot of viscosity versus rotational speed (rpm) was generated. The shape of the resulting curve provided insights into the rheological behavior of the gel formulation.^35^

####  Heat-cold cycle stability test

 To assess the stability of the formulations under temperature fluctuations, they were subjected to three cycles of temperature, 4 °C, ambient temperature and 40 °C. After each cycle, preparations were tested for any signs of phase separation, cracking, or other physical instability. Formulations that maintained their uniformity throughout the three cycles were considered stable.^33^

####  Assay of active ingredients

 The amount of both active ingredients in each prepared semi solid preparations, were measured accurately.

####  Release profile study

 The in vitro drug release profiles of the formulations were evaluated using two complementary methods as following description. Dialysis bag method in which predefined amount of each formulation was enclosed in a synthetic dialysis membrane (molecular weight cut-off: 14,000 Da) and immersed in an appropriate release medium under sink conditions and Franz diffusion cell method in which the receptor compartment of the Franz diffusion cell was filled with 50 mL of phosphate buffer (pH 6.8) containing 40% ethanol to ensure drug solubility. The formulation was applied to the donor compartment, and the system was maintained at 37 ± 0.5 °C with continuous magnetic stirring at 100 rpm. Aliquots (5 mL) were withdrawn from the receptor compartment at predetermined time intervals over a 24-hour period and replaced with an equal volume of fresh medium to maintain sink conditions. Skin permeability test which can provide more details is recommended for future studies, the presented study was focused on the evaluation of the clinical efficacy of prepared formulations and some in vitro characterizations were carried out before the application of the formulations. Samples collected from both methods were analyzed using UV-Vis spectrophotometry to quantify the amount of drug released. All experiments were performed in triplicate (n = 3), and results are presented as mean ± standard deviation

####  Antimicrobial activity assessment

 The antimicrobial activity of the optimized formulation was evaluated using the well diffusion method. Mueller-Hinton agar was used for *S. epidermidis*. Blood agar was used for *P. acnes*. Sterile media were prepared and poured into Petri dishes to solidify.

 Suspension of *S. epidermidis* and *P. acnes* was prepared at a concentration of 1.5 × 10^8^ CFU/mL. The entire surface of each agar plate was evenly inoculated with the respective bacterial suspension.

 Wells were aseptically created in the agar plates. An appropriate volume of the optimized formulation was aseptically dispensed into each well. Plates were incubated at 37 °C for 24 hours for *S. epidermidis* and 96 hours for *P. acnes* to allow for optimal microbial growth.

 The diameters of the zones of inhibition around each well were measured in millimeters using a ruler. The experiment was performed in triplicate to ensure reproducibility.

 Antimicrobial studies revealed that the mean inhibition zone diameters for *S. epidermidis* were 19 mm and 16 mm for the gel (C2) and emulgel (F2), respectively, which were significantly smaller than that of the clindamycin gel control (35 mm; *P* < 0.0001). No statistically significant difference was observed between the gel (C2) and emulgel (F2) formulations (*P*= 0.2756). Similarly, for *P. acnes*, the inhibition zones measured 24 mm and 22 mm for the gel (C2) and emulgel (F2), respectively, both of which were significantly smaller than that of the clindamycin gel (36 mm; *P* < 0.001). Again, no significant difference was detected between the gel (C2) and emulgel (F2) (*P* = 0.6793).

###  Clinical trial

 A single-blind, comparative clinical study was conducted involving 31 patients diagnosed with mild to moderate acne vulgaris. The study population comprised 13 females and 14 males, with a mean age of 23.96 years (range: 12–37 years).

 Inclusion criteria were as follows:

- Age ≥ 12 years
- Non-pregnant and non-lactating during the study period
- Provision of written informed consent
- No known hypersensitivity to herbal products, including clove oil and curcumin
- No use of systemic acne treatments within 30 days prior to enrollment
- No use of topical anti-acne therapies within 2 weeks prior to enrollment

Exclusion criterion included withdrawal of consent at any point during the study.

####  Study design

 Each participant received two gels including Gel 1 which was Clindamycin gel and Gel 2 that contains combination of clove oil and curcumin.

####  Treatment protocol

 To minimize confounding variables, each patient applied both gels to contralateral body sites affected by acne. One gel was applied on one side of his/her body surface with acnes and the other gel was applied in the other side of the body for him/her. So, many variables effects like genetic factors were limited to influence the results of the study.

 One group of volunteers were instructed to apply just clindamycin gel (G1) twice a day and Other groups received one time G1 and one time combination gel of clove oil and curcumin (G2) in a day. The use of any other anti-acne medications or products was prohibited during the study period. As our clinical study lacks placebo and the absence of randomization between gel types, randomized controlled trials in future work are recommended.

####  Follow-up

 A one-week follow-up phone call was conducted to assess for any adverse events.

 At four weeks, patients were evaluated based on their lesion counts also, patient-reported outcomes assessed through a standardized questionnaire.

####  Statistical methods

 The Kolmogorov-Smirnov test indicated non-normal distribution of all quantitative variables. Therefore, the Wilcoxon signed-rank test was used to compare pre- and post-intervention values within each group, and the Mann-Whitney U test was used to compare changes between groups.

 Significant reductions were observed in the sample group compared to baseline for the following: total acne lesion count (*P* = 0.000), comedone count (*P* = 0.005), papule count (*P*= 0.000), pustule count (*P* = 0.002), and ASI (*P* = 0.000).

 Between-group comparisons revealed significant differences in the reduction of total acne lesions, papules, and ASI (*P* = 0.000) in favor of the combination therapy group. No significant between-group differences were observed in the reduction of comedones (*P* = 0.375) or pustules (*P* = 0.062).

## Results and Discussion

###  Antimicrobial evaluation results

 Clove oil and curcumin d*emonstrated* antimicrobial activity against *P. acnes* and *S. epidermidis*. The zones of inhibition were recorded in Table 2.

**Table 2:** Antibacterial inhibition zones caused by active ingredients

		**Concentrations (v/v %)**	**Inhibition zone (mm) Disc diffusion method**	**Inhibition zone (mm) Agar diffusion method**
Clove oil	*S. epidermidis*	4	12	16
		2	11	13
		1	8	10.5
		0.5	7.5	9
		0.25	-	Grown
		0.125	-	Grown
		0.0625	-	Grown
		Control (-)	Grown	Grown
		Clindamycin	23	22
	*P. acnes*	2	16	22.5
		1	15	22
		0.5	14	21
		0.25	13	19.5
		0.125	Grown	12.5
		0.0625	Grown	Grown
		0.03125	Grown	Grown
		Control (-)	Grown	Grown
		Clindamycin	35	36
Curcumin	*S. epidermidis*	1000	13	17
		500	12	14
		250	Grown	10.5
		125	Grown	Grown
		62.5	Grown	Grown
		31.25	Grown	Grown
		Control (-)	Grown	Grown
		Clindamycin	23	22
	*P. acnes*	2000	12.5	-
		1000	12	16
		500	11	15
		250	9.5	12
		125	Grown	Grown
		62.5	-	Grown
		31.25	-	Grown
		Control (-)	Grown	Grown
		Clindamycin	35	36

###  Minimum inhibitory concentration (MIC)

 MIC of clove oil and curcumin for* P. acnes* was 0.25% (v/v) and 250 μg/mL for curcumin, respectively. MIC of clove oil and curcumin for *S. epidermidis* was 0.5% (v/v) and 500 μg/mL.

###  Minimum bactericidal concentration (MBC)

 MBC of clove oil and curcumin for* P. acnes* was 2.5% (v/v) and 4 mg/mL, respectively and for S.* epidermidis* was obtained in 4% (v/v) by clove oil and 4 mg/mL by curcumin.

###  Checkerboard assay

 Against *S. epidermidis*, the combination of clove oil and curcumin exhibited synergistic activity.

 Against *P. acnes*, the combination of clove oil and curcumin demonstrated either additive effects or no significant interaction.

###  Formulation development

 Based on the MBC values determined for both bacteria, gel and emulgel formulations were developed. Subsequent characterization and evaluation of these formulations were conducted.

###  Visual inspections

 All prepared gels and emulgels exhibited a clear, uniform appearance and were free of any discernible odor.

###  Evaluation of extrudability and spreadability

 The ease of extrusion from the container and the spreadability of the gels and emulgels were evaluated. Formulations containing 0.75% and 1% Carbomer 940 exhibited high consistency, resulting in difficulty in extrusion and poor spreadability on both the skin and glass plates. Formulations containing 0.5% Carbomer 940 demonstrated poor extrudability from the container due to their loose consistency. Formulations containing 0.6% Carbomer 940 demonstrated optimal extrudability and spreadability upon manual application.

###  Formulation optimization

 Formulation optimization was conducted to improve gel stability, appearance, and antimicrobial efficacy. Increasing propylene glycol concentration to 10% prevented dehydration during storage, while 5% titanium dioxide effectively masked the yellow color of curcumin. Additionally, incorporating 30% ethanol significantly enhanced antibacterial activity against *S. epidermidis*, likely due to improved solubility and synergistic effects with clove oil and curcumin. These modifications were applied to the final gel formulation.

###  Emulgel formulation optimization

 Emulgel formulation was optimized to enhance stability and aesthetic properties. Increasing propylene glycol concentration to 10% prevented dehydration during storage, while 5% titanium dioxide effectively masked curcumin’s yellow color. Varying LLP concentrations (7.5% vs. 10%) showed no notable difference in appearance. The final emulgel formulations were evaluated for the release profiles of clove oil and curcumin.

###  Stability in the heat-cold cycle

 Both the optimized gel and emulgel formulations demonstrated stability after undergoing three cycles of temperature fluctuations (refrigeration at 4°C, ambient temperature, and incubation at 40 °C). No significant physical changes, such as phase separation or cracking, were observed in formulations.

###  pH determination

 The pH of the gel was 6.09, and the pH of the emulgel was 5.86. Both values fall within the acceptable range for topical skin products (pH 4-6).

###  Emulgel type determination

 Water wash test: The emulgel was easily removed by washing with water, indicating an O/W emulsion.

 Methylene blue dye test: The addition of methylene blue dye to the emulgel resulted in a green color, confirming it as an O/W emulsion.

###  Assay of active ingredients

 All the results of the assay were in acceptable range of 90-110% of claimed amount.

###  In vitro Release

**Figure 1:**
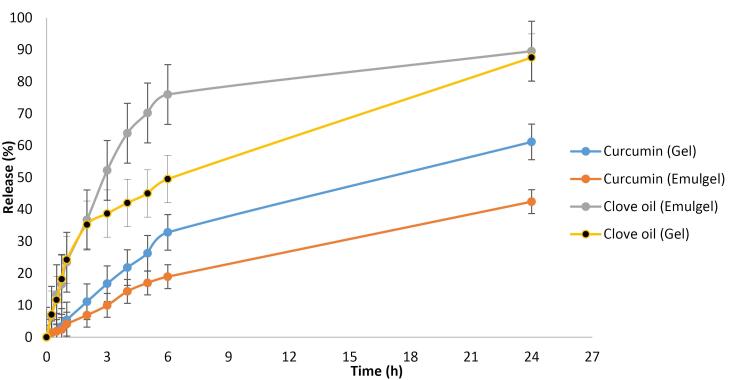
Release study graphs

 Franz diffusion cells were employed to evaluate the in vitro release profiles of the active ingredients. The results are presented in Figure 1.

###  Viscosity and rheological behavior

 Both gel and emulgel formulations exhibited non-Newtonian, shear-thinning behavior; the gel was thixotropic, while the emulgel showed stable, non-thixotropic flow.

###  Antimicrobial effect of desired formulations and clindamycin gel:

Table 3 presents the measured inhibition. Based on the observation of larger zones of inhibition exhibited by the gel formulation against both bacterial strains compared to the emulgel, the gel formulation was selected for subsequent clinical investigation.

**Table 3:** Comparison of antibacterial effects of designed formulations with clindamycin

**Type of bacteria**	**Formulation**	**Inhibition zone (mm)**
*S. epidermidis*	Gel (C2)	19
	Emulgel (F2)	16.5
	Clindamycin gel	35
*P. acnes*	Gel (C2)	24
	Emulgel (F2)	22
	Clindamycin gel	36

###  Clinical studies

Table 4 presents the demographic characteristics of the study participants. Twenty seven patients completed the study, while 4 were excluded due to factors such as non-adherence to the treatment regimen, missed follow-up appointments, or the concurrent use of other acne medications. The exclusion rate of 12.91% was considered negligible, with no significant impact on the overall study results or the validity of the conclusions.

**Table 4:** The demographic characteristics of the study participants

**Description**	**Number**	**Percent**
Age (y)		
≤ 20	9	33.3
21-30	15	55.6
31-40	3	11.1
Gender		
Male	14	51.9
Female	13	48.1
Drug history for acne		
No history	15	55.6
Topical	3	11.1
Oral	3	11.1
Topical & oral	6	22.2
Skin diseases history		
No history	11	40.7
Acne	13	48.1
Skin blemish	1	3.7
Skin allergy	1	3.7
Family skin diseases history		
No history	17	63
Acne	5	18.5
Skin blemish	2	7.4
Skin allergy	3	11.1

###  Statistical analysis

 Both treatment groups exhibited good tolerability during the 4-week study period. Minor side effects were observed: burning in four and burning-itching in one patient in the sample group within the first hour of application. In the positive control group, one patient reported dry skin. No other adverse events were noted during the study. The frequency of complications was comparable between groups based on chi-square analysis (*P* = 0.083). All observed side effects were mild and transient, resolving within two hours. Patient satisfaction with the drug formulation was 100% in both groups. Chi-square analysis revealed a statistically significant difference in patient-reported improvement between groups (*P* = 0.000), indicating higher satisfaction with the combination therapy (clove oil-curcumin gel and clindamycin gel) compared to clindamycin gel alone. Clinical outcomes demonstrated superior recovery in the combination therapy group. 62/96 patients in the clindamycin gel group and 100% of patients in the combination therapy group showed improvement. Among the 27 patients, 10 (27.04%) experienced excellent recovery, with 9 from the combination therapy group and 1 from the clindamycin gel group.

Tables 5-6 show the treatment groups and the therapeutic response of drug regimens for them. The clinical study of designed formulations was summarized in these tables.

**Table 5:** Treatment groups and responses to the therapeutic regimens

	**Clindamycin gel treatment regimen** **(mean ± Standard deviation)**		**Clove oil-curcumin gel+clindamycin gel treatment** **regimen (mean ± Standard deviation)**	
	**Before intervention**	**After intervention**	**Before intervention**	**After intervention**
Number of total acne lesions	11.25 ± 11.26	7.07 ± 8.72	15.11 ± 13.93	4.67 ± 6.18
	*P *value (comparing with before intervention) = 0.000		*P *value (comparing with before intervention) = 0.000	
Number of comedones	6.15 ± 10.95	4.78 ± 8.29	7.37 ± 13.46	3.15 ± 5.83
	*P *value (comparing with before intervention) = 0.011		*P *value (comparing with before intervention) = 0.005	
Number of acne papules	4.78 ± 3.18	2.26 ± 2.21	6.93 ± 3.98	1.52 ± 1.42
	*P *value (comparing with before intervention) = 0.000		*P *value (comparing with before intervention) = 0.000	
Number of pustules	0.33 ± 0.62	0.04 ± 0.19	0.81 ± 1.04	0 ± 0.00
	*P *value (comparing with before intervention) = 0.033		*P *value (comparing with before intervention) = 0.002	
Severity of acne according to ASI index	8.52 ± 6.33	4.72 ± 4.85	12.24 ± 8.07	3.093 ± 3.41
	*P *value (comparing with before intervention) = 0.000		*P *value (comparing with before intervention) = 0.000	

**Table 6:** Statistical analysis oftherapeutic responses to the drug regimen

	**The number of changes compared to before the intervention**	
	**Clindamycin gel**	**Clove oil-curcumin gel+clindamycin gel**
Number of total acne lesions	7.07-11.25 = -4.19	4.67-15.11 = -10.44
	*P* value (comparing between two groups) = 0.000	
Number of comedones	-1.37	-4.22
	*P* value (comparing between two groups) = 0.375	
Number of acne papules	-2.52	-5.41
	*P* value (comparing between two groups) = 0.000	
Number of pustules	-0.29	-0.81
	*P* value (comparing between two groups) = 0.062	
Severity of acne according to ASI index	-3.8	-9.15
	*P* value (comparing between two groups) = 0.000	

**Figure 2:**
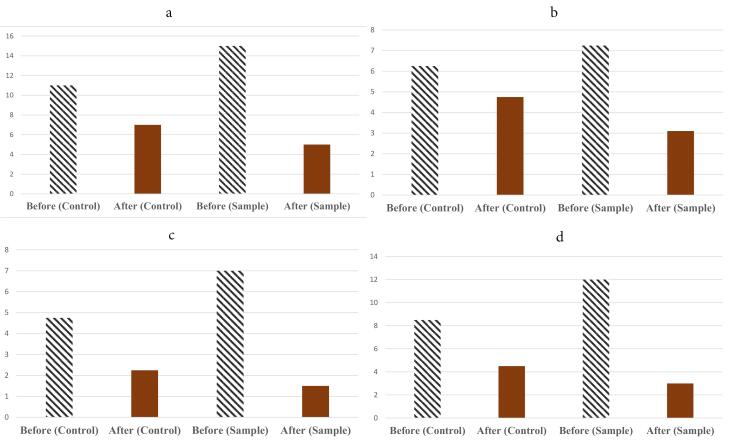
Efficacy results of prepared formulation (C2 Gel) on different symptoms including total acne lesions (a), comedones (b), pustules (c) and papules (d)

Figure 2 shows the efficacy results of prepared formulation (C2 Gel) on different symptoms.

 The following section discusses the above outcomes in the context of previous researches as well as potential clinical relevance. Acne vulgaris is a chronic inflammatory skin condition affecting the pilosebaceous units. Contributing factors include increased sebum production, follicular hyperkeratosis (blockage of the sebum secretion pathway), *P. acnes* overgrowth, and inflammatory processes. Studies suggest that IL-1β, Th2 cells, and IL-17 play a key role in acne-related inflammation.^24^ Furthermore, T lymphocyte accumulation around the follicle is observed during comedone formation, with *P. acnes* playing a primary role in developing inflammatory lesions. Mediators such as prostaglandins, macrophages, cytokines, and interleukins, particularly IL-1α, IL-1β, and IL-8, are critical contributors to the inflammatory response.^25,26^

 Alternative therapeutic approaches as we did in present study was attractive for researchers due to antibiotic resistance and possible teratogenic and other adverse health effects associated with conventional treatments. Isotretinoin is associated with significant adverse effects, including teratogenicity, with reported risks of 20–35% for major congenital anomalies.^36^

 Based on this concept Clove oil and curcumin have garnered increasing interest for their synergistic potential anti-bacterial and anti-inflammatory properties in acne treatment.

 Studies have demonstrated the bacteriostatic and bactericidal effects of clove oil against various bacterial strains, including *P. acnes* and *S. epidermidis*. Fu et al proposed a unique mechanism of action for clove essential oil, involving damage to the bacterial cell wall and inhibition of protein synthesis (kDa-18 and kDa-33) in *P. acnes*, ultimately leading to bacterial death.^11,12^ Moreover in vitro studies suggest that clove extracts or eugenol at non-toxic concentrations can significantly suppress the production of NF-mediated inflammatory factors (TNF-α, IL-1β, IL-6, and IL-8) and matrix metalloproteinase-9 by *P. acnes*-stimulated THP-1 cells, indicating potential for treating acne-induced skin inflammation.^14^ On the other side Saeed et al found that a clove oil emulsion exhibited anti-inflammatory activity in vivo, reducing inflammation and promoting wound healing in carrageenan-induced paw edema and incisional wound models in rats.^27^

 Misar et al reported that a formulation containing 1% clove oil displayed the strongest antimicrobial activity against acne-associated microorganisms, including *P. acnes, S. epidermidis*, *Staphylococcus aureus*, and *Candida albicans*.^28^ Liu et al demonstrated that a combination of 0.43 µg/mL curcumin and 17.2 µg/mL lauric acid could inhibit 50% of P. acnes growth, suggesting its potential application in treating *P. acnes*-related diseases.^16^ C. Xu et al revealed that *P. acnes* induces the secretion of inflammatory cytokines (TNF-α, IL-8, and IL-6) by monocytes and keratinocytes through the Toll-like receptor (TLR) pathway. They further demonstrated the anti-inflammatory effects of curcumin by downregulating TLR2 expression, inhibiting IL-6 production, and blocking TLR2-NF-κB signaling. A study by Zaman et al evaluated the antioxidant activity of a 5% turmeric rhizome methanolic extract cream formulation using the DPPH method. The study also investigated the effect of the cream on sebum secretion in 13 human volunteers using a sebumeter. The results indicated that the turmeric extract could potentially reduce sebum secretion in individuals with acne.^19^

 Sumit et al investigated liposomal gel formulations containing curcumin, lauric acid, azithromycin, and a combination of curcumin and lauric acid. The liposomal gel formulation with a 1:1 ratio of curcumin and lauric acid demonstrated the strongest antibacterial activity against *P. acnes* based on the agar diffusion method. In vivo studies using a mouse ear model revealed that the curcumin-lauric acid combination gel significantly reduced comedones and cytokine levels (TNF-α and IL-1β) compared to the placebo group.

 Alihosseini et al highlighted the potential role of turmeric and its active ingredient, curcumin, as a natural antioxidant in wound healing due to its ability to inhibit bacteria growth as well as healing agent.^37^ Previous studies indicates that herbal treatments can serve as effective alternatives or adjuncts to traditional therapies for both mild and moderate acne vulgaris.^38-40^ This study investigated the antimicrobial activity of clove oil and curcumin against *P. acnes* and *S. epidermidis*. Semi solid preparations were prepared and applied on the skin of volunteers suffering from acne, the synergic effect of the formulations with clindamycin gel was demonstrated.

## Conclusion

 Acne vulgaris is a chronic inflammatory condition involving multiple pathogenic factors including P. acnes, immune responses, and excessive sebum production. Concerns over antibiotic resistance and the side effects of conventional treatments like isotretinoin have led to growing interest in herbal alternatives.^37-44^ Clove oil and curcumin have demonstrated strong antibacterial and anti-inflammatory effects against *P. acnes* and *S. epidermidis*, through mechanisms such as disruption of bacterial membranes and suppression of pro-inflammatory cytokines. This study explored their combined efficacy in topical formulations, highlighting their potential as complementary therapies in acne management. The results indicate that the combination of clindamycin gel with a clove oil-curcumin formulation significantly reduced acne lesions, papule count, and ASI scores compared to clindamycin alone. These findings support the potential of this combination therapy as a more effective approach for acne management. Further large-scale, controlled clinical studies are needed to validate these outcomes.

## Competing Interests

 The authors declare no conflict of interest.

## Ethical Approval

 The study was approved by the Ethic Committee of Islamic Azad University. Code: IR.IAU.PS.REC.1400.212.

## Supplementary Files

Supplementary file contains Figures S1.
