# Supplementary figures and images for: Evaluating the Efficacy of a Semi-Solid Formulation with Clove Oil and Curcumin versus Clindamycin in the Treatment of Acne Vulgaris: A Comprehensive Study of Preclinical and Clinical Findings
Source: Adv Pharm Bull. 2025 Aug 30;15(3):606–16. doi: 10.34172/apb.025.45153 (PMC12703380; doi:10.34172/apb.025.45153)

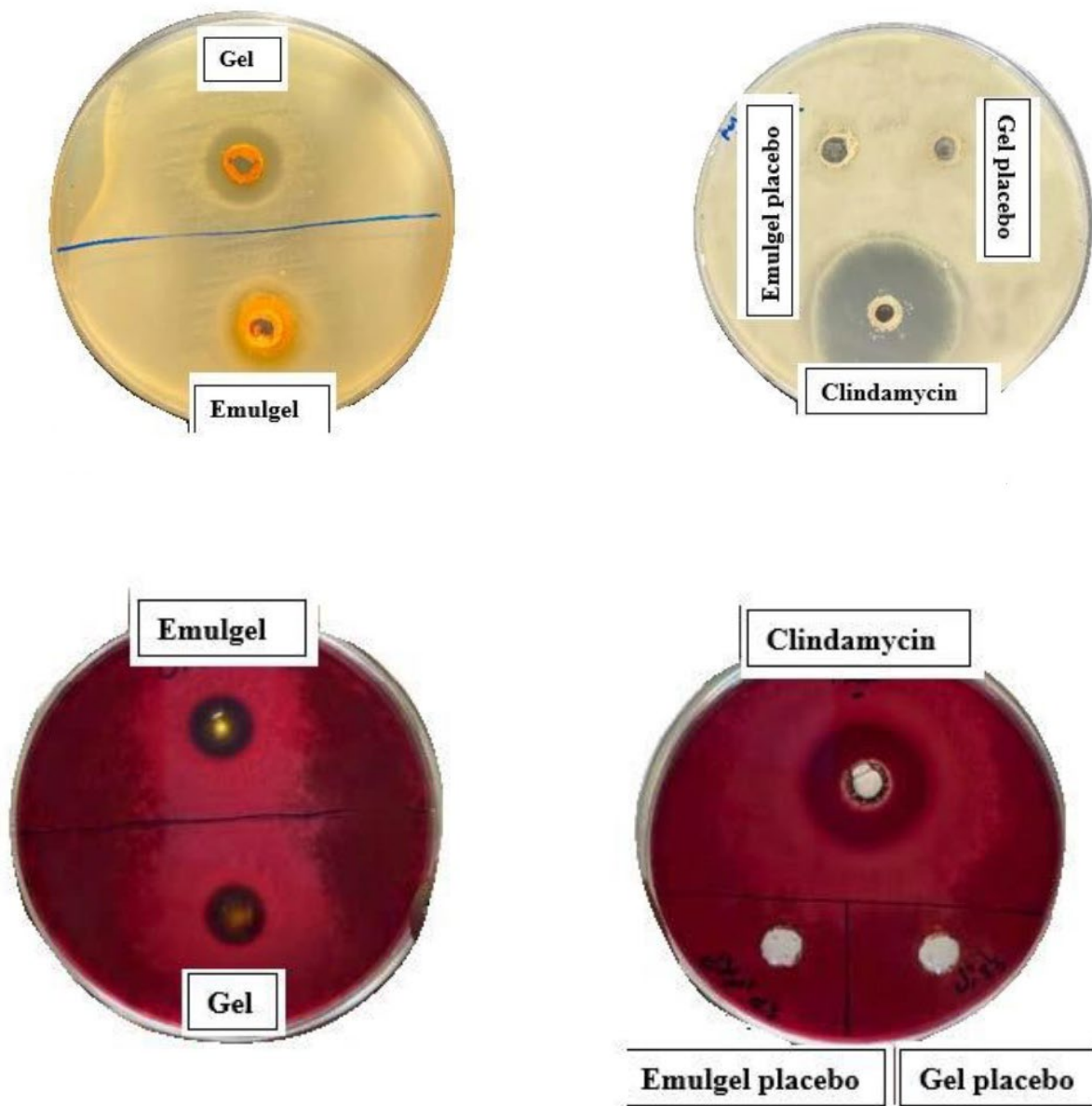

**Figure S1.** Antibacterial study results of desired formulations.

Supplement: Supplementary file 1 — Supplementary file contains Figures S1. [file apb-15-606-s001.pdf]
